# Reward and avoidance learning in the context of aversive environments and possible implications for depressive symptoms

**DOI:** 10.1007/s00213-019-05299-9

**Published:** 2019-06-28

**Authors:** Miriam Sebold, M. Garbusow, P. Jetzschmann, D. J. Schad, S. Nebe, F. Schlagenhauf, A. Heinz, M. Rapp, N. Romanczuk-Seiferth

**Affiliations:** 1 Department of Psychiatry and Psychotherapy, Charité – Universitätsmedizin Berlin, Charitéplatz 1, 10117 Berlin, Germany; 2 Department for Social and Preventive Medicine, University of Potsdam, Potsdam, Germany; 3 Cognitive Science, University of Potsdam, Potsdam, Germany; 4 Zurich Center for Neuroeconomics, Department of Economics, University of Zurich, Zurich, Switzerland; 5 Max Planck Institute for Human Cognitive and Brain Sciences, 04303 Leipzig, Germany

**Keywords:** Reward learning, Avoidance learning, Reinforcement learning, Computational psychiatry, Decision-making, Affective modulation, Depression symptoms

## Abstract

**Background:**

Aversive stimuli in the environment influence human actions. This includes valence-dependent influences on action selection, e.g., increased avoidance but decreased approach behavior. However, it is yet unclear how aversive stimuli interact with complex learning and decision-making in the reward and avoidance domain. Moreover, the underlying computational mechanisms of these decision-making biases are unknown.

**Methods:**

To elucidate these mechanisms, 54 healthy young male subjects performed a two-step sequential decision-making task, which allows to computationally model different aspects of learning, e.g., model-free, habitual, and model-based, goal-directed learning. We used a within-subject design, crossing task valence (reward vs. punishment learning) with emotional context (aversive vs. neutral background stimuli). We analyzed choice data, applied a computational model, and performed simulations.

**Results:**

Whereas model-based learning was not affected, aversive stimuli interacted with model-free learning in a way that depended on task valence. Thus, aversive stimuli increased model-free avoidance learning but decreased model-free reward learning. The computational model confirmed this effect: the parameter lambda that indicates the influence of reward prediction errors on decision values was increased in the punishment condition but decreased in the reward condition when aversive stimuli were present. Further, by using the inferred computational parameters to simulate choice data, our effects were captured. Exploratory analyses revealed that the observed biases were associated with subclinical depressive symptoms.

**Conclusion:**

Our data show that aversive environmental stimuli affect complex learning and decision-making, which depends on task valence. Further, we provide a model of the underlying computations of this affective modulation. Finally, our finding of increased decision-making biases in subjects reporting subclinical depressive symptoms matches recent reports of amplified Pavlovian influences on action selection in depression and suggests a potential vulnerability factor for mood disorders. We discuss our findings in the light of the involvement of the neuromodulators serotonin and dopamine.

**Electronic supplementary material:**

The online version of this article (10.1007/s00213-019-05299-9) contains supplementary material, which is available to authorized users.

## Introduction

In animals and humans, the appearance of a predator or other dangers typically promote withdrawal. These stimulus-induced avoidance responses are hard-wired and can be very beneficial in the presence of a mortal threat. Conceptually, holding back from threat reflects a Pavlovian response because a stimulus automatically elicits an action that is independent from the organism’s behavior. Pavlovian responses are disentangled from operant responses, where actions are elicited because they have repeatedly been paired with a specific consequence (reward or punishment).

Interestingly, interactions between Pavlovian avoidance responses and operant actions have been reported. Examples for these interactions are evident in real-life situations, as negative facial expressions reduce consumption behavior (Winkielman et al. 2005) and bad weather on election days reduces voter turnouts (Bassi 2013).

Experimentally, two types of such interactions have been reported: first, a stimulus-induced avoidance response can exaggerate another avoidance action. Consequently, negative stimuli increase an acquired withdrawal behavior, a phenomenon entitled as aversive Pavlovian-to-instrumental transfer (PIT) effect (Geurts et al. 2013b; Lewis et al. 2013; Nord et al. 2018). Second, stimulus-induced avoidance responses can interfere with approach behavior. Accordingly, negative stimuli decrease appetitive responses (Lee et al. 2005; Nelson and Sanjuan 2006), a phenomenon termed as conditioned suppression. Both of these phenomena provide evidence that negative valence can interact with actions. Further substantiation for these interactions come from studies showing impaired go but facilitated no-go responses when subjects anticipate punishment (Crockett et al. 2012a; Crockett et al. 2009; Guitart-Masip et al. 2012; Guitart-Masip et al. 2011).

Much attention has been drawn to phenomena where aversive stimuli interact with operant actions, mostly because such interactions also play a role in psychopathologies (Dayan et al. 2006). In mood disorders, for instance, patients are biased towards negative environmental stimuli and these biases amplify avoidance behavior (Eshel and Roiser 2010). Indeed, in depressive patients, aversive cues exert increased control over avoidance behavior (Nord et al. 2018).

So far, previous studies investigating valence-action interactions have realized actions along an axis of activation, thus as approach/avoidance or go/no-go responses. No study has so far investigated how aversive stimuli interacted with complex learning and decision-making, where subjects have to choose between several options in order to optimize their decisions. The analogy between complex decision-making and activation is at hand, as the former can also be mapped on a valence axis, ranging from avoidance- (minimize punishment) to reward- (maximize reward) based decision-making.

Importantly, recent computational accounts of complex learning and decision-making have emphasized the existence of two distinct control mechanisms, where actions can be guided by either model-free or model-based computations (Daw et al. 2011). Model-free controllers are reflexive and habitual and involve the retrospective updating of action values. Model-based controllers on the other side are adaptive and goal-directed and choose actions based on an internal model.

With regard to valence-action interactions, the concept of model-free control is specifically interesting because it shares many features of Pavlovian control including automaticity and rigidity (Friedel et al. 2014; Gillan et al. 2015). Moreover, model-free and Pavlovian control processes are assumed to rely on very similar neurobiological and computational mechanisms (Dayan and Berridge 2014).

Interestingly, certain internalizing pathologies such as depression have been associated with the dominance of the habitual system at the expense of the goal-directed system (Huys et al. 2015a; Huys et al. 2015b). In line with this, negative affective states such as acute stress shift decision-making away from model-based control, especially in subjects with depressive symptoms (Heller et al. 2018) or previous adverse life experiences (Radenbach et al. 2015).

The overarching aim of the present study was to elucidate the computational mechanisms of how aversive stimuli interacted with learning and decision-making. Given previous valence-action interactions (Guitart-Masip et al. 2014), we expected opposing roles on reward and avoidance learning. More precisely, we hypothesized impairments in reward learning, but enhancements in avoidance learning in the presence of negative stimuli. With regard to the computational mechanism of modulation, we asked whether model-free or model-based controllers would be affected by aversive stimuli.

As previous research has shown increased modulation of avoidance responses in the presence of aversive stimuli (Nord et al. 2018) as well as stress-induced disruptions in model-based control in depression (Heller et al. 2018), we further asked whether interactions between aversive influences on reward and avoidance learning were moderated by subclinical depression scores.

## Methods

### Subjects

Subjects were *n* = 55 21-year-old males, who had previously taken part in a larger study investigating learning in alcohol dependence (Lead study, ClinicalTrials.gov identifier: NCT01744834). All subjects were free from psychotropic medication and had no axis-1 psychiatric disorder as indicated by the Composite International Diagnostic Interview (CIDI, (Jacobi et al. 2013; Wittchen and Pfister 1997), see S1 for full inclusion criteria). In the Lead study, the 2-step task in its original form (no background stimuli, reward condition only) had previously been administered twice (Nebe et al. 2018). Therefore, subjects were familiar with the general task structure. Due to technical problems, the experiment was aborted in one subject, leaving a final sample of *n* = 54. Subjects were compensated for their participation with a fixed amount of 20 € plus an additional sum contingent on task performance (max. 15 €). Participants’ demographic and clinical characteristics are outlined in Table S1.

### Task

**Fig. 1:**
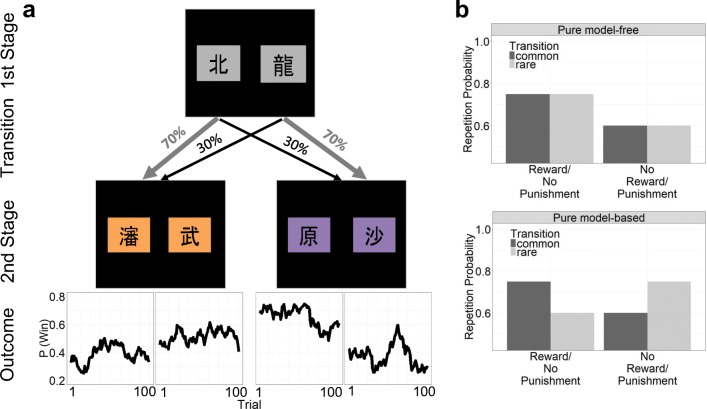
**a** Trial configuration: in each trial, subjects had to make two consecutive choices. At a first stage, subjects chose one stimulus over the other and then proceeded to a second stage where they chose between two stimuli. Second-stage choices were probabilistically rewarded or punished according to Gaussian random walks. Transition probabilities between first and second stages varied for first-stage choices: whereas one stimulus choice led commonly (70% of all trials) to one second-stage stimulus pair and rarely (30% of all trials) to the second-stage stimulus pair, the opposite was true for the other first-stage choice. **b** Expected stay probabilities of pure model-free and pure model-based learning

We used a two-stage Markov decision task with separate reward and punishment conditions as previously described (Worbe et al. 2016). Briefly, on each trial in the first stage, subjects made a choice between two stimuli, which led to one of two pairs of second-stage stimuli with fixed probabilities (70 and 30% of choices). Each of the four second-stage stimuli was associated with probabilistic 20 Euro Cent monetary reward in the reward condition and loss in the punishment condition or no monetary outcome (Fig.1a).

Model-free and model-based learning make distinct predictions about first-stage choice repetition probabilities (Fig. 1b). A model-free strategy predicts a higher repetition probability of first-stage choices that led to reward in the previous trial, therefore predicting a main effect of outcome on first-stage repetitions. However, a model-based strategy predicts the incorporation of the transition frequency of the previous trial and therefore the interaction between outcome and transition on first-stage repetitions. For instance, it predicts lower repetition probabilities when the transition frequency of the previous trial was rare but rewarded, because the unchosen first-stage stimulus has a higher probability in leading to the rewarding second-stage stimulus pair. The same is true for the other task valence when choices lead to no punishment.

**Fig. 2:**
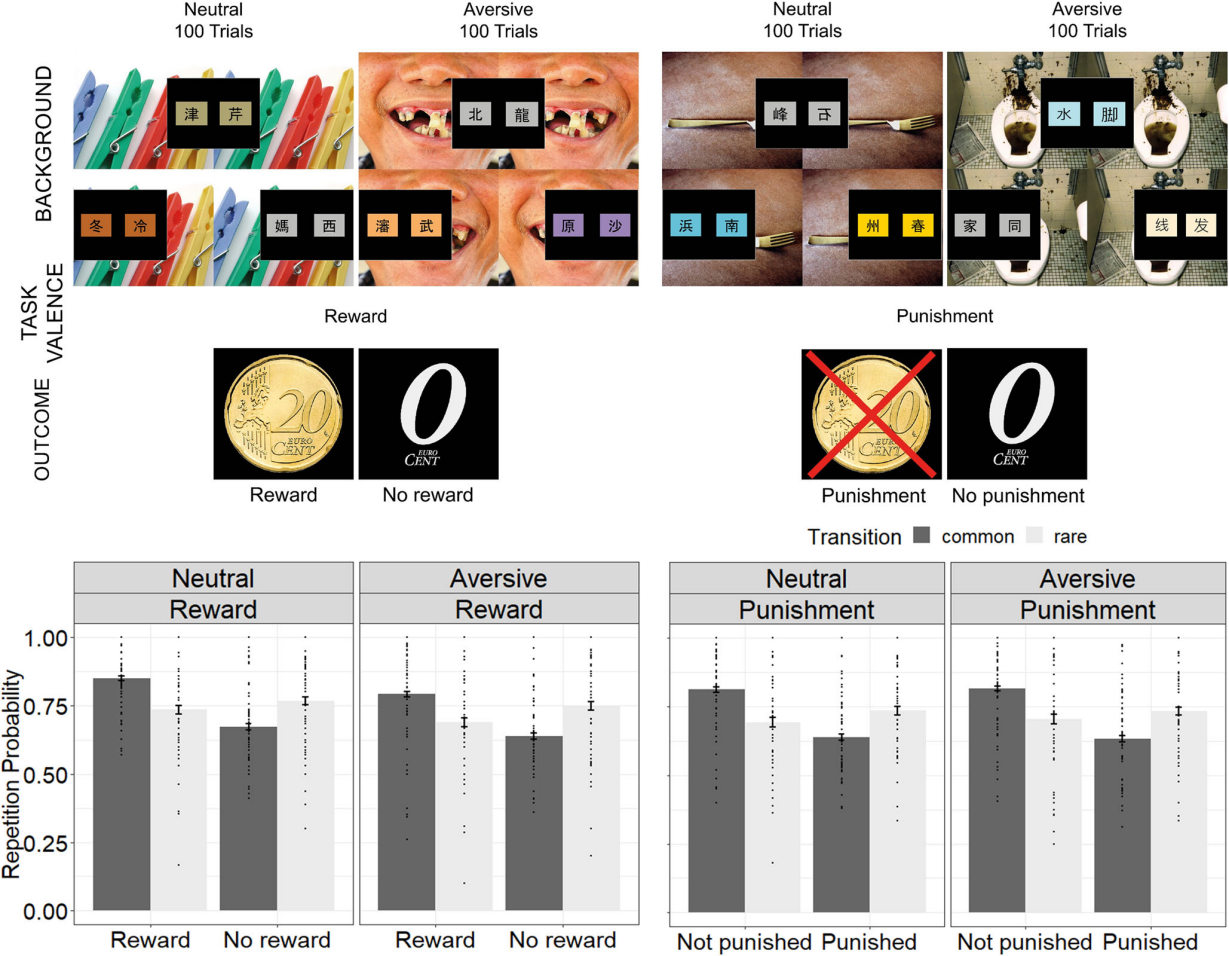
**a** Schematic presentation of the task. Subjects performed 100 trials within each condition (neutral reward, aversive reward, neutral punishment, aversive punishment). Stimulus sets and courses of second-stage reward probabilities (random walks) varied between all conditions. First and second stages were presented consecutively and are presented simultaneously here for illustrative purposes of the different stimulus sets. **b** Overall performance: across all conditions, subjects showed a mixture of model-free and model-based behavior (main effect outcome and transition × outcome interaction)

Reward and punishment conditions were either deposited with neutral or aversive background stimuli (Fig. 2), resulting in a 2 (background) × 2 (task valence) within-subject design (neutral reward, aversive reward, neutral punishment, aversive punishment). Each background stimulus was shown four times to fill the entire screen, as previously done in human PIT paradigms (Garbusow et al. 2016; Garbusow et al. 2014). The background stimulus appeared at the beginning of each trial and stayed there during the complete two-step trial (first, second stage, and outcome).

All four conditions of the task had the same transition probabilities and dynamic range of the reward or punishment probability. The courses of these second-stage outcome probabilities (random walks) were slightly different between all four conditions but were randomly assigned to each condition. All conditions had different color code and stimuli set on the first and second task stages. The used sets were identical to Liu et al. (2016). Participants completed 100 trials for each condition divided into two sessions. The order of the reward and punishment conditions as well as the order of the aversive or neutral background conditions was counterbalanced across subjects.

Neutral and aversive background stimuli were drawn from the international affective picture system (IAPS, Lang et al. 2005). Picture selection was based on the ratings of the male college cohort as reported in Lang et al. (2008). To exclude pictures in the aversive condition with potential disruptive or harmful content, we limited our selection to pictures with ratings of moderate arousal. Our final aversive image selection included pictures with lowest ratings in valence and lower arousal ratings than the mean + 0.2 standard deviations. Our final neutral image selection included pictures with lowest arousal ratings and valence ratings between mean valence of the cohort and the mean + 0.2 standard deviations.

Based on visual inspection, we excluded pictures with black borders, sexual content, and duplicates. Each condition consisted of a final set of 50 pictures (see Table S2). Each picture was shown twice, once in the reward and once in the punishment condition. Examples of stimuli in the neutral set are household articles such as a tissue, a basket, or a lamp whereas the aversive set includes images of contaminated toilets, sick or dead animals, and violence. Post hoc comparisons of the ratings by Lang et al. (2008) of our final stimulus set (using Wilcoxon rank sum test) indicated that pictures of the aversive condition were rated as more negative (*p* < 0.0001) and more arousing (*p* < 0.0001) than neutral pictures.

Before the experiment, all subjects underwent the self-paced computer-based instructions explaining the structure of the task and providing examples. Overall, the subjects were instructed to win as much money as they could in the reward condition and to avoid monetary loss in the punishment condition of the task. Participants were told that they would be paid for the experiment depending on their cumulative performance in the reward and the punishment condition. After an extensive training session, subjects were confronted with 3 multiple choice questions on the structure and the reward versus punishment condition of the task (adapted from Gillan et al. (2016)). Subjects only advanced to the experiment if they answered all questions correctly.

### Questionnaire

In a separate session which was conducted prior to the experimental session, subjects answered (among other questionnaires) the German version of the Hospital anxiety and depression scale (HADS, Herrmann-Lingen 2005; Zigmond and Snaith 1983), which includes 14 items, of which 7 items are related to anxiety symptoms and 7 items are related to depressive symptoms. Subjects can respond to each item on a scale between 0 (do not agree at all) and 3 (fully agree). Scores are summed up and can vary between 0 and 21 for both subscales. Previous studies have found that depressive symptoms increase the impact of negative states on decision-making (Heller et al. 2018). Moreover, negative Pavlovian stimuli decreased approach but increased avoidance responses in depressive patients, but not in matched healthy controls (Nord et al. 2018). Based on these findings, we explored the association between subclinical depressive symptoms and task behavior on an explorative basis.

### Analyses

We performed two sets of analyses. The first was a mixed effects logistic regression where first-stage choices (stay/switch) were regressed on the previous trial outcome (reward/no reward or no punishment/punishment), transition frequency (common/rare), the background (aversive/neutral), and task valence (reward/punishment). Within-subject factors (intercept, main effect of outcome, main effect of transition, main effect of background, task valence and their interaction) were taken as random effects across participants. For visual purposes, we extracted model-free and model-based scores as previously described (Sebold et al. 2014). Briefly, model-free scores reflected the individual main effect of outcome on first-stage repetition probabilities (% reward/not punished common + % rewarded/not punished rare − % unrewarded/punished common − % unrewarded/punished rare), whereas model-based scores reflected the interaction between transition frequency and outcome on first-stage repetition probabilities (% reward/not punished common + % unrewarded/punished rare − % rewarded rare/not punished − % unrewarded/punished common). As we were specifically interested in how aversive stimuli alter these scores depending on task valence, we calculated these scores between aversive and neutral conditions for the reward and the punishment condition respectively (separate for model-free and model-based control).

The second analysis was the fit of the original Daw et al. (2011) reinforcement learning model (7-parameter hybrid model, see S4) to the data. We also fitted two reduced models for model comparison and selection (see S5). We used an expectation maximization algorithm to find maximum a posteriori estimates. We fitted the model simultaneously for all four conditions and compared parameters of interest. Practically, the model consists of two different sets of parameters: the reinforcement learning parameters that capture the internal learning and evaluation processes and the response (softmax) parameters that associate the result of the internal valuations to choices. We hypothesized that background and task valence would specifically influence learning parameters but had no hypothesis on how it would affect softmax parameters. The learning parameters in the hybrid model included two learning rate parameters (α1 and α2 for first and second stages, respectively); the weighting parameter *ω*, which indicates the balance between model-free and model-based control; and the eligibility trace parameter *λ* from the model-free algorithm, which indicates how much second-stage outcomes update first-stage action values.

These four reinforcement parameters (α1, α2, *ω*, *λ*; for their distribution, see Table S3) were subjected to a multivariate 2 × 2 ANOVA analysis with background and task valence as within-subject effects and tested for interactions. Post hoc tests included univariate 2 × 2 ANOVAs with background and task valence as repeated measure factors for all four parameters.

A key scientific question of reinforcement learning concerns how accurate RL fits actually are. Indeed, using rather complex algorithms can lead to ambiguous or false parameter identifications that mischaracterize participants actual learning strategies. One way to evaluate this is to generate simulation data from the computational model with the parameters that have been estimated from the real data. In an ideal world, the simulation data should faithfully match the real data (Gureckis and Love 2009). Thus, we ran additional simulation analyses, where individual first- and second-stage actions were generated from the computational model with the previously estimated parameters. For each subject, we computed 100 simulation data sets for each condition (neutral reward, aversive reward, neutral punishment, aversive punishment). Consequently, we compared model-free (main effect outcome) and model-based (interaction between outcome and transition) scores between the real data and the simulated data. Moreover, we tested for interaction effects between task valence, outcome, background, and transition in the simulated data.

Regression analyses were conducted using generalized linear mixed-effects models implemented with the lme4 package (Bates et al. 2015) in the R programming language, version 3.1.2 (cran.us.r-project.org). Computational modeling was performed in Matlab 2014 (8.3., 2014a). MANOVA for modeling parameters was computed using the RM function from the MANOVA.RM package (Friedrich et al. 2017) in R.

## Results

### Behavioral data

Subjects missed comparably few trials (mean = 6.5, sd = 7.4 trials). Twenty-six subjects began with the punishment condition, and 28 subjects began with the reward condition. Moreover, 20 subjects began with aversive and 34 began with the neutral background stimuli, suggesting that the randomization was effective.

The logistic regression indicated a main effect of outcome (*p* < 0.0001), a main effect of transition frequency (*p* < 0.05), and an interaction between outcome and transition frequency (*p* < 0.0001). Thus, subjects showed a mixture between model-free and model-based learning (Fig. 2b), as previously shown in this cohort (Nebe et al. 2018).

**Fig. 3:**
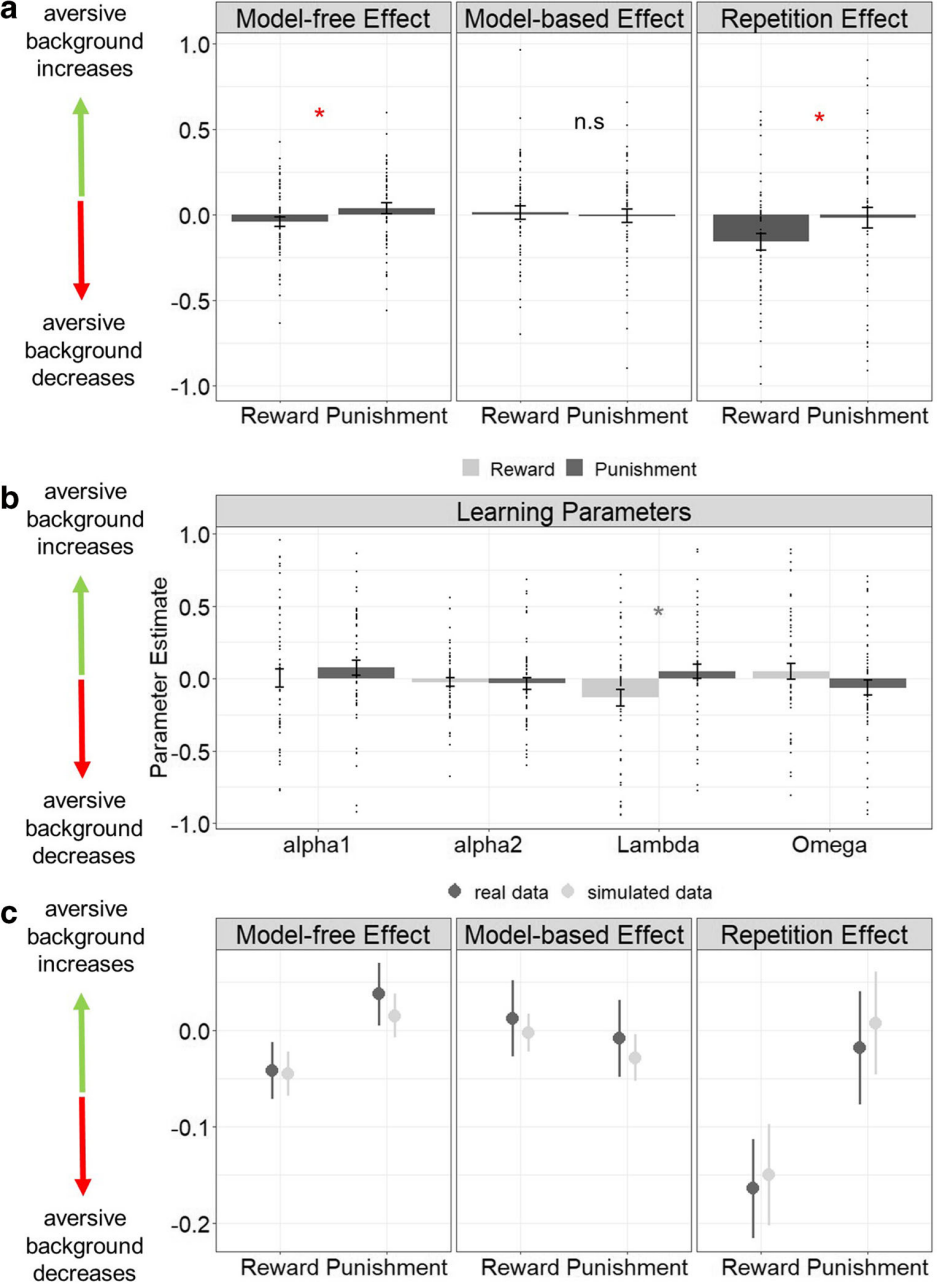
Results of how aversive stimuli interacted with model-free and model-based reward and punishment learning and first-stage repetition, which indicates stochasticity or exploration behavior, independent of learning. Bar plots indicate means and standard errors of means for the difference scores between aversive versus neutral background condition **a Left:** aversive background enhanced model-free learning in the punishment condition, but reduced model-free learning in the reward condition. **Middle:** aversive background did not influence model-based learning in the reward or punishment condition. **Right:** aversive background selectively decreased first-stage repetition behavior in the reward condition. **b** The stage-skipping update parameter *λ* was differently influenced by aversive background in the reward and punishment condition. Other learning parameters were not affected by aversive background in the reward or punishment condition. **c** Behavioral data simulations from the extracted parameters captured the observed reinforcement-dependent effect of aversive background on model-free learning (**left**) and the repetition effect (**right**) and replicated the null finding regarding model-based learning (**middle**)

Neither task valence (*p* = 0.1) nor background stimuli per se *(p* = 0.2) influenced choice behavior. However, we found an interaction between task valence and background (*p* = 0.011), suggesting that aversive stimuli influenced first-stage repetition behavior depending on task valence (Fig. 3a, right panel). Post hoc analyses indicated that aversive stimuli decreased repetition in the reward condition (*p* = 0.002), but not in the punishment condition (*p* = 0.41).

Importantly, the interaction between background and task valence was further modulated by outcome, as indicated by the three-way interaction of outcome × task valence × background (*p* = 0.041). This result indicates that aversive stimuli affect model-free learning in a way that depends on task valence, justifying further post hoc analyses. These post hoc analyses indicated an interaction between outcome × task valence in the presence of aversive background stimuli (*p* = 0.04), but not neutral background stimuli (*p* = 0.44), indicating that aversive stimuli promoted model-free learning in the punishment condition, but decreased model-free learning in the reward condition (Fig. 3a, left panel). The interaction between transition frequency and outcome was not modulated by task valence (*p* = 0.6), background (*p* = 0.9), or the interaction between task valence and background (*p* = 0.9) indicating that model-based performance was not modulated by either of the two manipulations (Fig. 3a, middle panel). Complete results of the regression analysis can be found in Table 1 (real data).

**Table 1:** Fixed effects results from the mixed effects logistic regression, where stay behavior on first stages was regressed on previous trial’s outcome × transition × task valence × background. Significant main effects and interactions are displayed in italics. Effects are displayed for the measured data (left column) and the simulated data from the computational modeling parameters (right column). Effects of the simulated data match observed effects from the measured data

	Real data		Simulation data	
	Estimate	*p* value	Estimate	*p* value
*Intercept*	*1.191*	*< 0.0001*	*1.259*	*< 0.0001*
*Outcome*	*0.432*	*< 0.0001*	*0.547*	*< 0.0001*
*Transition*	*0.121*	*0.019*	*0.160*	*< 0.0001*
Task valence	0.072	0.092	0.170	0.062
Background	0.087	0.152	0.131	0.061
*Outcome × transition*	*1.403*	*< 0.0001*	*1.279*	*< 0.0001*
Task valence × transition	− 0.510	0.546	0.041	0.178
Outcome × task valence	− 0.059	0.466	0.021	0.803
Transition × background	− 0.002	0.983	− 0.008	0.689
Outcome × background	0.005	0.947	0.124	0.105
*Task valence × background*	*0.329*	*0.011*	*0.379*	*0.034*
Transition × outcome × task valence	0.014	0.595	0.205	0.170
Transition × outcome × background	0.013	0.947	0.086	0.459
Transition × task valence × background	0.011	0.942	− 0.036	0.437
*Outcome × task valence × background*	*0.314*	*0.041*	*0.320*	*0.018*
Transition × outcome × task valence × background	− 0.047	0.898	0.020	0.928

### Computational modeling

The MANOVA indicated a significant interaction between background and task valence (ANOVA-type statistic (ATS) = 2.78, *p* = 0.042). Post hoc univariate tests showed no difference for the balance of model-free and model-based control *ω* (*F*_(1,159)_ = 2.27, *p* = 0.14), for first-stage learning rates α1 (*F*_(1,159)_ = 0.84, *p* = 0.36) nor for second-stage learning rates α2 (*F*_(1,159)_ = 0.03, *p* = 0.86), but significant differences in stage-skipping update *λ* (*F*_(1,159)_ = 4.73, *p* = 0.03, Fig. 3b). The parameter *λ* signifies a stronger influence of reward prediction errors at the second stage on first-stage decision values and accounts for the main effect of outcome observed in first-stage stay behavior. In line with raw data analysis, this speaks for a subtle, albeit significant, elevation of model-free learning in the presence of aversive stimuli in the punishment condition, but impairments in the reward condition. Explorative comparison of parameters of the softmax decision model (β1, β2, *ρ*) showed no significant modulation by task valence or background (ANOVA-type statistic (ATS) = 1.39, *p* = 0.249, see Fig. S1).

Simulation analyses confirmed the observed effect, as, overall, subjects showed decreased model-free learning in the aversive environment in the reward condition, but increased model-free learning in the aversive environment in the punishment condition (Fig. 3C, left panel). Moreover, in line with the real data, model-based learning was not influenced by task valence and aversive environments (Fig. 3c middle panel). Simulation analyses further demonstrated that the aversive environment decreased the repetition effect on first stages in the reward condition (Fig. 3c, right panel). Moreover, we found high correlations between model-free scores of the real data and model-free scores of the simulated data (*ρ* = 0.71, *p* = < 0.0001) and similarly high correlations between model-based scores of the measured data and model-based scores of the simulated data (*ρ* = 0.7, *p* = < 0.0001). When performing the linear mixed effects logistic regression with the simulated data, we again found a 2-way interaction between task valence and background (*p* = 0.034) and a 3-way interaction between outcome, task valence, and background (*p* = 0.018), suggesting that our model parameters indeed captured the observed effects (Table 1, simulation data).

### Association with subclinical depression scores

**Fig. 4:**
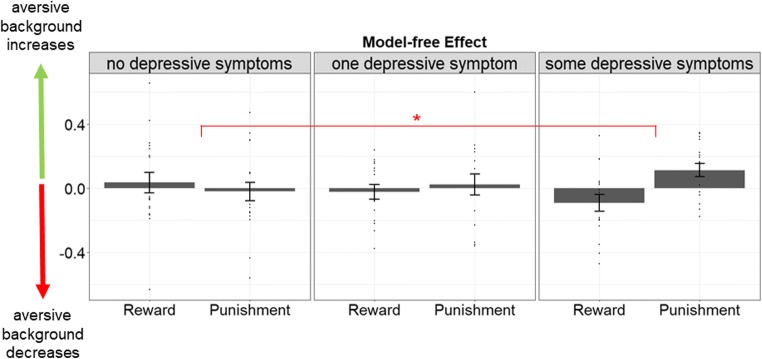
Exploratory analysis comparing subjects at the two ends of a depression score showed that mainly subjects with some depressive symptoms showed aversive background influences of aversive background stimuli on model-free learning depending on the task valence

We further wanted to test the association between the observed influence of aversive stimuli on model-free learning in the punishment and reward condition and interindividual variation in subclinical depression scores. In accordance with the fact that none of the subjects met the criteria for major depression, subjects scored very low on the HADS subscale. Most subjects stated that they had no depressive symptoms at all (*n* = 19), whereas 16 subjects reported one depressive symptom and 17 subjects reported two or more depressive symptoms. For exploratory analyses, we tested whether the extreme ends of this distribution (none vs. some depressive symptoms) would differ in the observed effect. Therefore, we performed the abovementioned regression analysis with an additional between-subject factor, indicating the occurrence of depressive symptoms. Indeed, this regression analysis revealed a 4-way interaction between the observed effect and the presence of depressive symptoms (task valence × outcome × background × group, *p* = 0.032, Fig. 4a). Post hoc analyses where interactions were separately tested for both groups indicated that only subjects with mild depressive scores showed valence-dependent influences of the background on model-free learning (task valence × outcome × background, *p* = 0.002), whereas this effect was absent in subjects with no depressive symptoms (task valence × outcome × background, *p* = 0.772). There was no interaction between group, task valence, and background (*p* = 0.938) indicating that modulation of first-stage repetition by task valence and background was not affected by depressive symptoms (Fig. 4b).

## Discussion

Here, we show that aversive context stimuli influence complex learning and decision-making in a way that depends on task valence. We found that aversive stimuli increased model-free learning when subjects had to avoid punishments but decreased model-free learning when subjects had to approach rewards.

This result is in accordance with a number of studies demonstrating valence-action interactions in the negative domain, as facilitation of avoidance responses have been reported in the presence of aversive stimuli (Campese et al. 2017; Dickinson and Pearce 1977). Moreover, our finding matches reports about aversive Pavlovian-to-instrumental transfer (PIT) effects in humans (Geurts et al. 2013a; Lally et al. 2017; Nord et al. 2018; Rigoli et al. 2012), where conditioned aversive stimuli decrease operant responses. The finding of our study extends these previous reports and shows that aversive stimuli do not only interact with rather simple approach/avoidance responses but also with more complex learning and decision-making processes. This is particularly important given the fact that humans are often required to make challenging decisions in various emotional states or environments.

According to a recent computational account, complex learning and decision-making can be dichotomized along two control systems, a habitual model-free system and a goal-directed model-based system (Daw et al. 2011). The here applied two-step task enables to disentangle the relative contribution of these control systems. Interestingly, in our study, only model-free, but not model-based learning, was modulated in a valence-dependent way by aversive stimuli.

This finding is particularly interesting given the large debate on whether valence-action interactions as seen in PIT relate to model-free or model-based control mechanisms (Dayan and Berridge 2014). One recent study showed that aversive PIT in humans reflected habitual rather than goal-directed behavior (Garofalo and Robbins 2017), which speaks to an association between model-free mechanisms and PIT. Although our procedure was fundamentally different from the PIT procedure, it shares one feature, that is, Pavlovian control over operant responding. Pavlovian and model-free control mechanisms are assumed to be innately specified. Interestingly, bodily freezing—a major innately specified defensive responses to threat (Blanchard et al. 2001)—is associated with Pavlovian control over operant responding (Ly et al. 2014), which speaks to a subtle (although no direct) evidence that Pavlovian control over operant behavior relates to the model-free system. Although, in our study, aversive stimuli did not impact model-based decision-making, some other studies have shown that negative states, such as acute stress (Heller et al. 2018; Otto et al. 2013), decrease model-based control (but see Gillan et al. 2019). One possible explanation for the discrepant results is the qualitative different manipulation types (acute stress vs. aversive stimuli) which might tax different control mechanisms.

A computational model substantiated our findings of a valence-dependent effect of aversive stimuli on model-free control. The computational parameter lambda, which codes influences of second-stage reward prediction errors on first-stage action values, was increased under aversive influences in the punishment condition but decreased in the reward condition. No other learning parameters were affected by task valence and background.

Beyond learning, we found an interaction between task valence and background on choice behavior which was independent from learning. More precisely, in our study, aversive environments increased switch behavior in the reward condition. Increased first-stage switch behavior could either reflect exploration behavior, stochasticity, or noise, and unfortunately, the two-step task does not enable to disentangle these components. However, two parameters from the softmax could potentially have mirrored the effect: β1, indicating how first-stage choices are guided by expected values and the repetition parameter ρ, indicating general stickiness. None of these parameters was modulated by task valence and environment. We believe that the failure of the computational model to capture the effect from the regression analysis relates to a power problem as the large variance within the softmax parameters makes it harder to detect between condition variations. However, despite the failure of the computational model to replicate the repetition effect, our simulation analyses demonstrated the ability of the model to predict all raw data results, which shows that the model could recover all behavioral effects.

Our computational model further provides a mechanistic framework on how aversive stimuli affect reward and avoidance learning. A famous biologically based computational model proposes distinct dopaminergic cortico-striatal pathways for both types of learning (Frank et al. 2004). According to this model, dopamine bursts increase synaptic plasticity in the direct pathway and thereby support reward learning whereas dopaminergic dips reduce synaptic plasticity in the indirect pathway through which avoidance learning is strengthened. Crucially, reductions in tonic dopamine levels, as seen in Parkinson’s disease, lead to increased avoidance learning but disrupted reward learning, a functional status that is reversed through dopaminergic medication (Frank 2005; Frank et al. 2004). Interestingly, this finding is in line with our data of increased model-free punishment learning but decreased model-free reward learning in the presence of aversive stimuli. As it seems unlikely that aversive pictures change individual’s tonic dopamine levels, one might speculate that aversive stimuli alter phasic dopaminergic activity, e.g., increase dopaminergic dips induced by punishment (model-free negative prediction errors) and decreased reward-related dopaminergic bursts (model-free positive prediction errors). Future studies should investigate this hypothesis in animals using microdialysis or single cell recordings.

Interestingly, exploration behavior (which might be reflected by the here observed effect of increased switching during reward learning with aversive background) has also been associated with tonic dopamine. L-DOPA selectively increased first- (Wunderlich et al. 2012) and second- (Kroemer et al. 2019) stage switch behavior in the reward version of the two-step task and remediated task switching problems in Parkinson patients (Cools et al. 2001). Studies investigating the catechol-O-methyltransferase (COMT) gene further support a model in which exploration has a dopaminergic basis (Frank et al. 2009; Kayser et al. 2015). Whereas the mentioned studies suggest a positive association between dopamine and exploration, one recent study suggested the opposite direction of this association as dopaminergic blockade in rats led to increases of random exploration behavior (Cinotti et al. 2019). Thus, future studies should further investigate the association between dopamine and the influence of negative states on exploration behavior.

With regard to interindividual differences, our exploratory analyses showed that valence-dependent effects of how aversive stimuli affect model-free learning were only evident in subjects reporting mild, subclinical symptoms of depression, but not in subjects with no symptoms in depression. This effect is in line with studies demonstrating increased aversive PIT effects in depression (Nord et al. 2018) and matches a recent study that reports increased influences of negative states on complex decision-making in subjects with depressive symptoms (Heller et al. 2018).

The dysfunctional serotonergic system in depression (Belmaker and Agam 2008; Graeff et al. 1996; Mann 1999) might relate to these altered influences of aversive stimuli on learning and decision-making. Evidence comes from studies using tryptophan depletion to lower central 5-HT levels (Crockett et al. 2012b). Some studies have shown that tryptophan depletion lowers the impact of aversive stimuli (Crockett et al. 2012a) or expected punishments (Crockett et al. 2012a; Crockett et al. 2009; den Ouden et al. 2015) on inhibition and decreases aversive PIT effects (Geurts et al. 2013b), suggesting that low serotonin reduces the influence of aversive stimuli on actions. However, increases in the influence of aversive stimuli on response inhibition have also been reported under tryptophan depletion (Cools et al. 2008; Hebart and Glascher 2015). Moreover, two studies have shown that tryptophan depletion impairs model-based reward learning (Worbe et al. 2015) while it enhances model-based avoidance learning (Worbe et al. 2016), suggesting that low serotonin may have valence-dependent effects. Moreover, in line with the assumption that low serotonin induces negative biases, genetic studies show that subjects carrying the less functional s allele in the promoter region of the serotonin transporter gene (5-HTTLPR) show increased avoidance learning (Finger et al. 2007) and amplified neural responses to aversive stimuli (Canli et al. 2005; Hariri et al. 2005; Heinz et al. 2005). In the current study, we did not assess central serotonin levels or genetic variations of the serotonergic transporter gene and future studies should further investigate the interactions between serotonin deficiency, depressive symptoms, and emotional biases of decision-making to elucidate potential vulnerability markers for depression.

Our study has several limitations: first, the statistical evidence for our observed effects are tentative. The targeted interaction effects in this study are of higher order (4- and 5-fold interactions), and large sample sizes are usually needed to detect these kinds of effects. Additional analyses where we tested the hypothesis that our observed effects were stronger for model-free than for model-based control (see S3) failed to reach statistical significance. Therefore, although we have direct evidence that model-free control was affected by task valence and background, we cannot conclude that our effects were statistically stronger for model-free compared with model-based control. As this might again be a power problem, our findings ultimately need replication in bigger sample sizes.

Moreover, we used negative IAPS pictures that were rated as more negative in valence, but also as more arousing compared with neutral pictures. Therefore, we cannot rule out that the observed effects were due to arousal instead of valence. The arousal system has been closely linked to fight or flight reactions (Roelofs 2017), and a recent study has shown that arousal but not valence of stimuli affects approach behavior (Bouman et al. 2015). Moreover, arousal as indexed by pupil diameters is positively related to exploration (Jepma and Nieuwenhuis 2011), which is in line with our observation on increased switching behavior (potentially indexing exploration) in aversive compared with neutral environments.

However, two findings speak against the arousal hypothesis. First, a u-shaped relationship between arousal and reaction times has been reported, where moderate and high arousal decreases and increases RTs, respectively (Nishisato 1966; Welford 1971). Second, an inverted u-shape association between arousal and task performance has been reported (Broadhurst 1957; Neiss 1988), where moderate and high arousal increases and decreases performance respectively. Model-based control is the more sophisticated control system in the two-step task and might be related to task performance as it correlates with processing speed and working memory capacity (Schad et al. 2014). As we used moderately arousing pictures, we could have expected decreases in first-stage RTs but increased model-based control. However, aversive environments neither affected reaction times (see S2) nor model-based control, which speaks against the arousal hypothesis. The additional use of positive, highly arousing pictures would allow ruling out the alternative arousal instead of valence explanation. Due to time restriction, we could not implement this additional manipulation in the current experiment.

One second limitation of the study is the extremely homogenous sample (male 21-year-old subjects), which limits the generalizability of its findings. Also, because we only included subjects with no current axis-1 psychiatric disorder, the distribution of the depression score was heavily skewed with a strong zero inflation. Beyond this, one further limitation was that depressive symptoms were assessed in a separate session prior to the assessment of the task. A recent study on almost 200 young adults (Jinnin et al. 2017) showed that mild depressive symptoms remained stable over a 1-year follow-up period, which makes us believe that this could also hold for this sample. However, further studies that aim at investigating depression vulnerability should sample subjects from a high-risk population, e.g., first-degree relatives of depressive patients.

Besides these limitations, our study is the first to provide a computational model of how negative environmental stimuli influence complex decision-making. Thus, these results are of high interest for a better understanding of context-specific effects on learning mechanisms in everyday life. Further, our study adds a mechanistic model of how alterations of these effects can be understood as a potential vulnerability marker for depression.

## Electronic supplementary material

ESM 1 (DOCX 255 kb)
