# Supplementary material for: Reward and avoidance learning in the context of aversive environments and possible implications for depressive symptoms
Source: Psychopharmacology (Berl). 2019 Jun 28;236(8):2437–49. doi: 10.1007/s00213-019-05299-9 (PMC6695365; doi:10.1007/s00213-019-05299-9)
Supplement: Supplementary file 1 — (DOCX 255 kb) [file 213_2019_5299_MOESM1_ESM.docx]

# **Electronic Supplementary Information**

Title: Reward and avoidance learning in the context of aversive environments and possible implications for depressive symptoms

Journal: Psychopharmacology

Authors: Sebold, M.^1,2^, Garbusow, M.^1^, Jetzschmann, P.^1^, Schad, D.^2^, Nebe, S.^3^, Schlagenhauf, F.^1^, Heinz, A.^1^, Rapp, M.^2^, Romanczuk-Seiferth, N.^1^

^1^ Department of Psychiatry and Psychotherapy, Charité Campus Mitte (CCM), Charité - Universitätsmedizin Berlin, Berlin, Germany,

^2^ Department for Social and Preventive Medicine, University of Potsdam, Potsdam, Germany,

^3^ [Laboratory for Social and Neural Systems Research](https://www.researchgate.net/institution/University_of_Zurich/department/Laboratory_for_Social_and_Neural_Systems_Research-SNS_Lab), University of Zürich, Switzerland

Corresponding Author:

Miriam Sebold

Charité Universitätsmedizin Berlin

Department of Psychiatry and Psychotherapy

Address: Charitéplatz 1, 10117 Berlin, Germany

Phone: +49 30 450 517257

E-Mail: [miriam.sebold@charite.de](mailto:miriam.sebold@charite.de)

## Method S1: Participants’ inclusion criteria and characteristics

Participants who had taken part in the original study (Lead), were invited for this study. Study participation was approximately 3 years after the original study participation. In the original study, subjects were randomly sampled from the population of 18-year-old men of two German cities (Berlin and Dresden) by the respective local registration office. Exclusion criteria of the original study were a history of or current neurological or mental disorders (except for nicotine dependence and alcohol abuse), left-handedness, and contra-indications for MRI. Participants had to have normal or corrected-to-normal vision. Because the original study investigated alcohol drinking trajectories, an additional inclusion criterion for participants was that they had at least two drinking occasions in the past 3 months.

Table S1: Demographic and clinical characteristic of the final sample (n = 54)

|  | **Mean (n)** | **SD** |
| --- | --- | --- |
| Education Years | 11.57 (54) | 1.27 |
| Verbal IQ (MWT-B, Lehrl (2005)) | 26.36 (53) | 4.24 |
| Cognitive Speed (DSST, Ryan and Lopez (2001)) | 10.68 (53) | 3.16 |
| Anxiety (HADS, Zigmond and Snaith (1983)) | 2.00 (52) | 1.91 |
| Depression (HADS Zigmond and Snaith (1983)) | 1.19 (52) | 1.25 |
| Impulsivity (BIS-15, Patton et al. (1995)) | 29.00 (53) | 4.66 |

***MWT-B:*** *Mehrfachwahl-Wortschatz-Intelligenztest,* ***DSST:*** *Digit Symbol Substitution Test,* ***HADS:*** *Hospital Anxiety and Depression Scale,* ***BIS-15:*** *Short German version of the Barrat Impulsiveness Scale 11*

Table S2: Final list of background stimuli in the experiment

|  | **Aversive Stimuli** | | **Neutral Stimuli** | | |
| --- | --- | --- | --- | --- | --- |
|  | Picture Content | IAPS Number | Picture Content | | IAPS Number |
| **1** | Hospital | 2205 | Fork | 7080 | |
| **2** | Cemetery | 9220 | Flowers | 5731 | |
| **3** | SadChild | 2800 | Window | 7490 | |
| **4** | DeadDog | 9185 | Mushroom | 5510 | |
| **5** | Garbage | 9340 | Runner | 8465 | |
| **6** | Garbage | 9295 | Teenager | 2870 | |
| **7** | DeadCows | 9181 | Mushroom | 5530 | |
| **8** | Cow | 9140 | Scarves | 7205 | |
| **9** | Bum | 2750 | PicnicTable | 7026 | |
| **10** | Cat | 9571 | Mushroom | 5520 | |
| **11** | Cigarettes | 9830 | Candlestick | 7053 | |
| **12** | Toilet | 9301 | Man | 2357 | |
| **13** | Baby | 2053 | NeutChild | 2270 | |
| **14** | Accident | 9435 | NeuMan | 2102 | |
| **15** | BatteredFem | 3181 | Shopping | 2745 | |
| **16** | Cemetery | 9000 | Bridge | 7547 | |
| **17** | Mud | 9031 | Boy | 2273 | |
| **18** | HungMan | 9265 | GirlMakeup | 2308 | |
| **19** | Toilet | 9302 | Adult | 2020 | |
| **20** | KidCry | 2301 | Butcher | 2235 | |
| **21** | Smoke | 9280 | Woman | 2372 | |
| **22** | ElderlyWoman | 2590 | Venusflytrap | 5040 | |
| **23** | Garbage | 9290 | Building | 7500 | |
| **24** | Handicapped | 9415 | Clothespins | 7052 | |
| **25** | Vomit | 9320 | Tools | 7019 | |
| **26** | Cigarette | 9831 | Couple | 2390 | |
| **27** | CryingFamily | 2456 | Bed | 7710 | |
| **28** | SickKitty | 9561 | Coffeecup | 7057 | |
| **29** | Pollution | 9342 | Bird | 1616 | |
| **30** | Dishes | 9395 | Mushroom | 5531 | |
| **31** | Exhaust | 9090 | Outlet | 6150 | |
| **32** | Teeth | 9043 | Golf | 8312 | |
| **33** | Garbage | 9291 | Puzzle | 7061 | |
| **34** | Cigarettes | 9832 | AbstractArt | 7237 | |
| **35** | SadGirls | 2455 | Satellite | 5471 | |
| **36** | Kids | 2278 | Bridge | 7546 | |
| **37** | Horses | 9182 | Mushrooms | 5532 | |
| **38** | Ticket | 9417 | Checkerboard | 7182 | |
| **39** | Firefighter | 9912 | Feet | 2445 | |
| **40** | Cemetery | 9001 | Building | 7242 | |
| **41** | ScaredChild | 9041 | HermitCrab | 1935 | |
| **42** | DentalExam | 9584 | Male | 2107 | |
| **43** | Accident | 9610 | Boat | 5395 | |
| **44** | DrunkDriving | 2751 | Turtle | 1945 | |
| **45** | BurntBldg | 9471 | Shrimp | 1903 | |
| **46** | ManInPool | 2055 | Buffalo | 1675 | |
| **47** | Pollution | 9341 | Agate | 7830 | |
| **48** | Roaches | 1275 | Fish | 7484 | |
| **49** | Attack | 6561 | Cowboy | 2635 | |
| **50** | CryingBoy | 2457 | AbstractArt | 7247 | |

## Method S2: Additional Reaction Time Analyses

We wondered whether increased switching behavior (interaction effect of task valence and environment on first stage repetition, see Fig. 3A, right column) would reflect vigor. Altered reaction times (RTs) of first stage choices could potentially reflect this. Therefore, we performed additional analyses, where we regressed first stage reaction times on task valence (Reward vs. Punishment) and Background (aversive vs. neutral). Within-subject factors (intercept, main effect of background, task valence and their interaction) were taken as random effects across participants. Neither Valence (p = .69), Background (p = .46), nor it’s interaction (p = .65) influenced first stage Reaction times. Thus, although choices seemed to be influenced by these factors, general vigor (which might be an index for arousal) wasn’t.

## Method S3: Additional Analyses: Is model-free over model-based control affected by task valence * background

We further tested if aversive stimuli influenced valence dependent learning stronger in the model-free than in the model-based control system. Therefore, we performed a difference score between model-free and model-based task modulated effects (indicating stronger influences of Task valence and Background on the model-free than the model-based system). We then conducted a one-sample t-test on the resulting variable to test significantly deviations from zero. This analysis failed to reach statistical significance (p = .14). Therefore, cannot conclude that model-free over model-based control was affected by background * task valence.

## Method S4: Computational model

The algorithm of the 7-parameter hybrid model includes both model-based and model-free subcomponents, which allows for mapping state-action pairs to expected future values.

The model-free strategy is computed using the SARSA (λ) temporal difference learning. At each stage *i* of each trial *t,* the value for each state-action pair was calculated as follows:

*Q_TD_ (s_i,t_,a_i,t_) = Q_TD_ (s_i,t_,a_i,t_) + α_i_ δ_i,t_*

where *δ_i,t_ = r_i,t_ + Q_TD_ (s_i+1,t_ , a_i + 1,t_) - Q_TD_ (s_i,t_ ,a_i,t_)* and *α_i_*  is a free learning parameter. Different learning rates *α_1_* and *α_2_* for the two task stages were estimated. The reinforcement eligibility parameter (λ) determines the update of the first-stage action by the second-stage prediction error as follows: *Q_TD_ (s_1,t_ , a_i1,t_) = Q_TD_ (s_1,t_ ,a_1,t_) + a_1_λδ_2,t ._*

The model-based reinforcement-learning algorithm was computed by mapping state-action pairs to a transition function and assuming that participants choose between two possibilities, as follows: *P(S_B_ ⏐S_A_, a_A_) = 0.7, P(S_C_ ⏐S_A_, a_B_) = 0.7* for common and

*P(S_B_ ⏐S_A_, a_A_) = 0.3 P(S_C_ ⏐S_A_, a_B_) = 0.3* for rare transitions,

where S is the state (first stage: S_A_; second stage: S_B_ and S_C_), and a is the action (two actions: a_A_ and a_B_) at a given state. The action value (Q_MB_) was computed at each trial from the estimates of the transition probabilities and outcomes and was defined for the first stage as follows:

*Q_MB_ (s_A_,a_i_) = P(s_B_|s_A_,a_i,_) max_a_ Q_TD_(s_B_,a) + P(s_C_|s_A_,a_i,_) max_a_ Q_TD_(s_C_,a)*

Finally, to connect values to choices, the weighted sum of the model-free and model-based values was computed for the first stage as defined:

*Q_net_(s_A_,a_j_) = w Q_MB_(s_A_,a_j_) + (1-w) Q_TD_(s_A_,a_j_)*

where *w* is the weighting parameter. Assuming that two approaches coincide at the second stage, and that Q_MB_ = Q_TD_, at the second stage Q_net_= Q_MB_ = Q_TD._ Then, the probability of a choice is the softmax equation for

Q_net_:

*Σ_a’_ exp (β_i_[Q_net_(s_i,t_,a) + p * rep(a)])*

*P(a_i,t_ = a|s_i,t_) = exp (β_i_[Q_net_(s_i,t_,a) + p * rep(a)]) /*

where the free inverse temperature parameters (β_i_) control the choice randomness, and *p* captures perseveration (p > 0) or switching (p < 0) in the first-stage choices. In total, the model contains 7 free parameters (β1, β2, α1, α2, λ, p, ω), with special cases of pure model-based (ω = 1) and model-free (ω = 0) models.

Table S3: Inferred mean parameters (standard deviations) for the full computational model. Parameters were estimated simultaneously for all four conditions (Reward Neutral, Reward Aversive, Punishment Neutral and Punishment Aversive)

|  | **Reward**  **Neutral** | | **Reward Aversive** | **Punishment Neutral** | **Punishment Aversive** |
| --- | --- | --- | --- | --- | --- |
| alpha 1 | 0.51 (0.33) | | 0.52 (0.35) | 0.42 (0.29) | 0.49 (0.30) |
| alpha 2 | 0.53 (0.29) | | 0.51 (0.25) | 0.56 (0.23) | 0.53 (0.27) |
| beta 1 (log) | | 1.80 (0.75) | 1.53 (1.27) | 1.64 (0.94) | 1.41 (1.24) |
| beta 2 (log) | | 1.15 (1.20) | 1.04 (1.06) | 0.80 (1.13) | 1.06 (0.81) |
| Lambda | 0.60 (0.34) | | 0.47 (0.36) | 0.53 (0.35) | 0.58 (0.34) |
| Omega | 0.47 (0.30) | | 0.52 (0.30) | 0.53 (0.32) | 0.47 (0.30) |
| Repetition | 0.17 (0.23) | | 0.20 (0.40) | 0.18 (0.35) | 0.15 (0.40) |


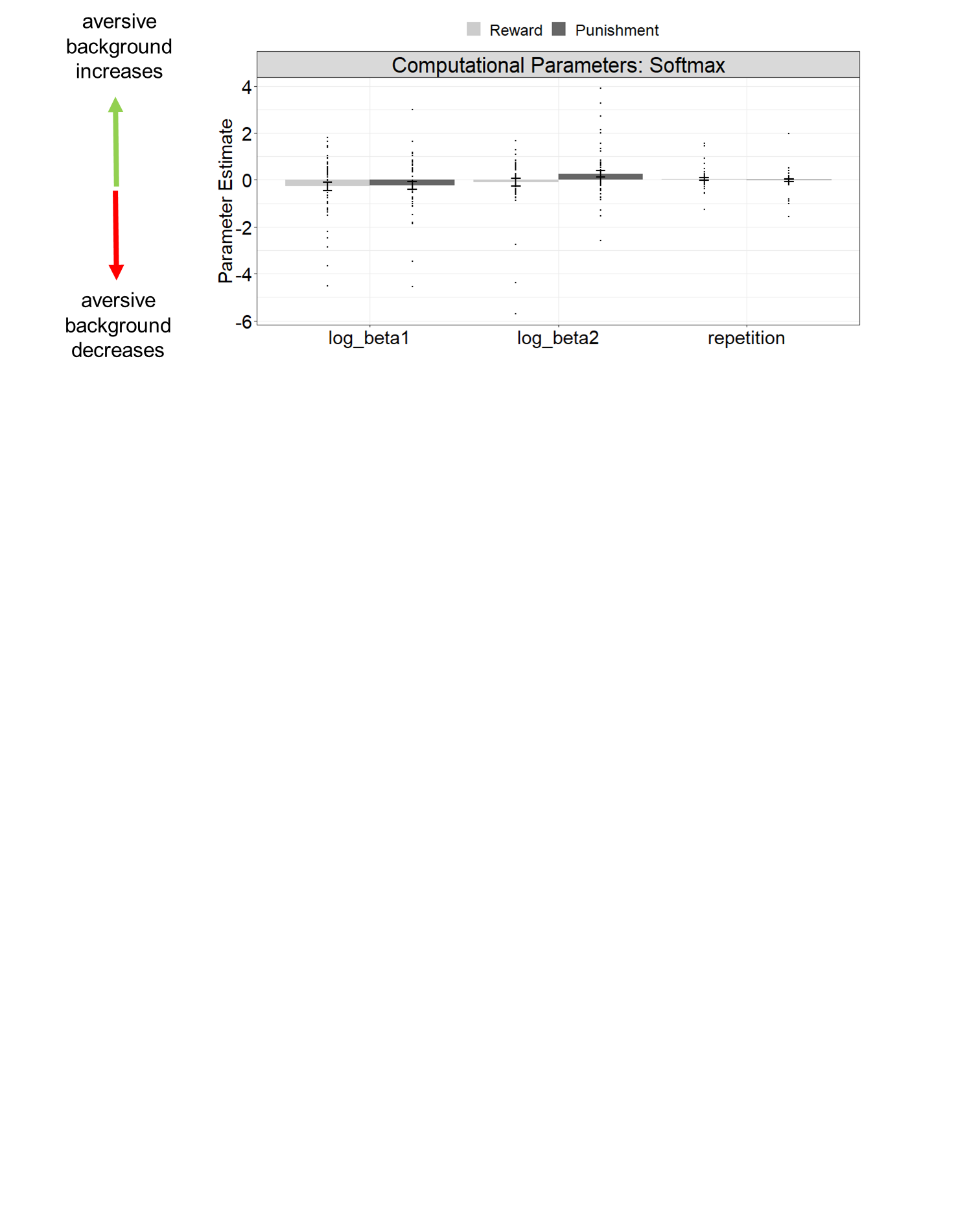


Figure S1: Valence dependent affective modulation of the Softmax parameters. None of the parameters were modulated by task valence and background.

## Method S5: Model selection procedure

We fitted two alternative models to our choice data: 1) a model-free algorithm SARSA (λ), which only captures a main effect of outcome on first stage choices, and 2) a pure model-based algorithm, which considers the interaction between outcome and transition frequencies, but does not capture a main effect of outcome on first stage choices. We subjected individual model evidences (negative log-likelihoods) for all three models to a between-conditions random effects Bayesian model selection procedure (Rigoux et al. 2014; Stephan et al. 2009) to compare model-fits across conditions (Reward Neutral, Reward Aversive, Punishment Neutral, Punishment Aversive). Because of the factorial task structure, we tested model selection along two factors, task valence (reward vs. punishment) and background (aversive vs. neutral). In line with previous studies (Sebold et al. 2017), the hybrid model was the best fitting model. This was not influenced by task valence or background stimuli (Figure S2).


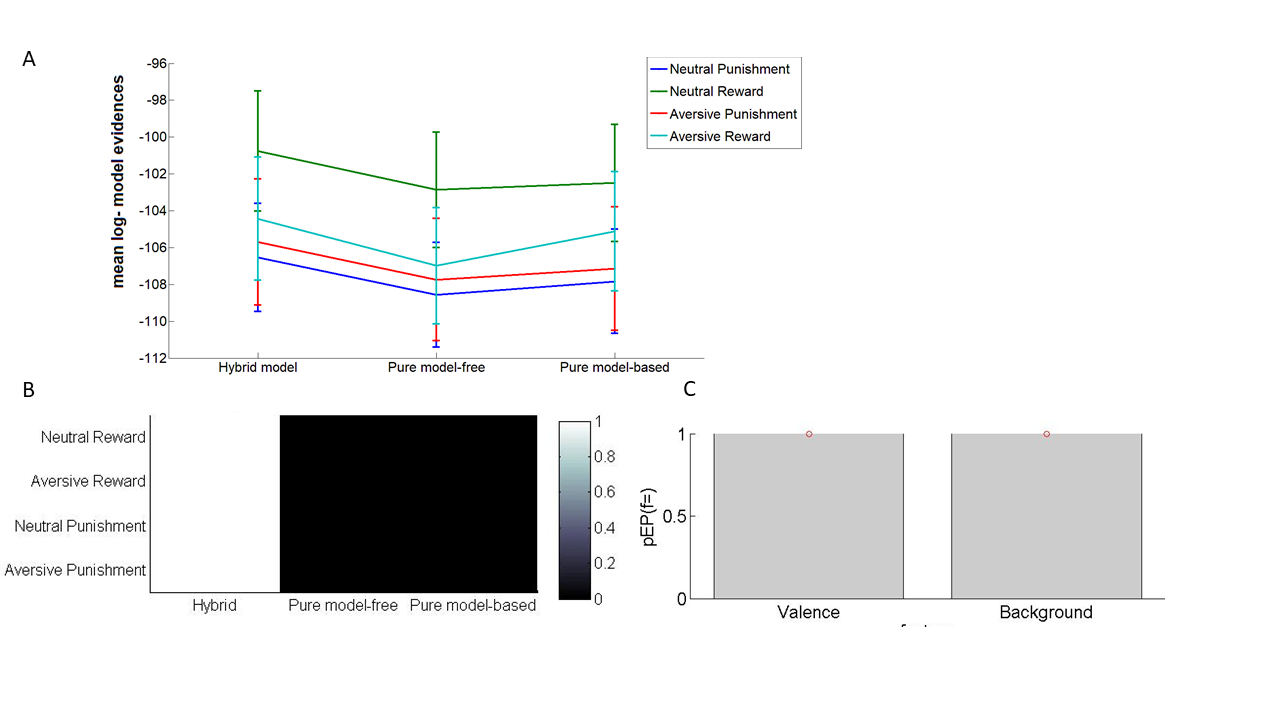


Figure S2: A) Under all conditions, the hybrid model yielded the best model evidence, as indexed by A) the mean negative log likelihood over each model, B) Per-condition Bayesian model selection: exceedance probability. C) Between-condition stability: protected exceedance probabilities.

## Literature

Lehrl S (2005) Mehrfachwahl-Wortschatz-Intelligenztest : MWT-B ; Manual zum MWT-B. Spitta-Verl., Balingen

Patton JH, Stanford MS, Barratt ES (1995) Factor structure of the Barratt impulsiveness scale. J Clin Psychol 51:768-774

Rigoux L, Stephan KE, Friston KJ, Daunizeau J (2014) Bayesian model selection for group studies - revisited. Neuroimage 84:971-985 doi:10.1016/j.neuroimage.2013.08.065

Ryan JJ, Lopez SJ (2001) Wechsler Adult Intelligence Scale-III. In: Dorfman WI, Hersen M (eds) Understanding Psychological Assessment. Springer US, Boston, MA, pp 19-42. doi:10.1007/978-1-4615-1185-4_2

Sebold M et al. (2017) When Habits Are Dangerous: Alcohol Expectancies and Habitual Decision Making Predict Relapse in Alcohol Dependence. Biol Psychiatry 82:847-856 doi:10.1016/j.biopsych.2017.04.019

Stephan KE, Penny WD, Daunizeau J, Moran RJ, Friston KJ (2009) Bayesian model selection for group studies. Neuroimage 46:1004-1017 doi:10.1016/j.neuroimage.2009.03.025

Zigmond AS, Snaith RP (1983) The hospital anxiety and depression scale. Acta Psychiatr Scand 67:361-370
